# Testing the effectiveness of genetic monitoring using genetic non‐invasive sampling

**DOI:** 10.1002/ece3.8459

**Published:** 2021-12-27

**Authors:** Anthony James Schultz, Kasha Strickland, Romane H. Cristescu, Jonathan Hanger, Deidre de Villiers, Céline H. Frère

**Affiliations:** ^1^ Global Change Ecology Research Group University of the Sunshine Coast Sippy Downs Qld Australia; ^2^ Icelandic Museum of Natural History (Náttúruminjasafn Íslands) Reykjavik Iceland; ^3^ Department of Aquaculture and Fish Biology Hólar University Hólar Iceland; ^4^ Endeavour Veterinary Ecology Pty Ltd Toorbul Qld Australia; ^5^ School of Biological Sciences University of Queensland St Lucia Qld Australia

**Keywords:** degradation, koala, monitoring, non‐invasive, population genetics, simulation, SNP

## Abstract

Effective conservation requires accurate data on population genetic diversity, inbreeding, and genetic structure. Increasingly, scientists are adopting genetic non‐invasive sampling (gNIS) as a cost‐effective population‐wide genetic monitoring approach. gNIS has, however, known limitations which may impact the accuracy of downstream genetic analyses. Here, using high‐quality single nucleotide polymorphism (SNP) data from blood/tissue sampling of a free‐ranging koala population (*n* = 430), we investigated how the reduced SNP panel size and call rate typical of genetic non‐invasive samples (derived from experimental and field trials) impacts the accuracy of genetic measures, and also the effect of sampling intensity on these measures. We found that gNIS at small sample sizes (14% of population) can provide accurate population diversity measures, but slightly underestimated population inbreeding coefficients. Accurate measures of internal relatedness required at least 33% of the population to be sampled. Accurate geographic and genetic spatial autocorrelation analysis requires between 28% and 51% of the population to be sampled. We show that gNIS at low sample sizes can provide a powerful tool to aid conservation decision‐making and provide recommendations for researchers looking to apply these techniques to free‐ranging systems.

## Graphical abstract

Genetic non‐invasive sampling is a powerful tool for monitoring free‐ranging populations. Here we test the accuracy of using non‐invasively sampled DNA for downstream individual and population level analysis. We find that non‐invasive genetic sampling can provide accurate genetic measures, depending on the questions being investigated.

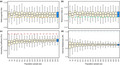

## 1 INTRODUCTION

Ongoing habitat loss and fragmentation (Dirzo et al., 2014) is forcing many terrestrial species into small, isolated populations (Manel & Holderegger, 2013). Such populations are at increased risks of inbreeding depression and genetic erosion. As such, genetic monitoring can inform habitat rehabilitation and corridor revegetation programs to re‐establish gene flow, and genetic interventions like genetic rescue to supplement genetic diversity or decrease inbreeding (Frankham, 2015; Weeks et al., 2011), among others. However, a lack of accurate information on genetic structure and health status of populations (particularly for rare or endangered species), makes conservation decision‐making a difficult task (Lynch & Lande, 1993; Willi & Hoffmann, 2009), and gathering such genetic information can be costly. Furthermore, despite some uptake of conservation genetics/genomics approaches into conservation practice, a substantial gap still exists (Shafer et al., 2015). Improved understanding of both how genetic approaches can benefit conservation decision‐making, and how to design and implement such studies into practical conservation, can have positive impacts on conservation outcomes. However, direct genetic monitoring may currently be inaccessible for hard‐to‐survey or non‐charismatic species with limited funding availability (Colléony et al., 2017).

The rise of genetic non‐invasive sampling (gNIS)—where DNA is extracted from animal scat, feathers, or hair (Waits & Paetkau, 2005)—has the potential to facilitate cost‐effective, accessible, large‐scale species identification and genetic monitoring (Ferreira et al., 2018). Applications of this method range from estimating population sizes and monitoring the genetic integrity of reintroduced populations to investigating natal philopatry and roosting behaviors. Similarly, environmental DNA approaches (eDNA), where genetic material is extracted from environmental samples such as soil or water, can be used for species identification and monitoring applications (Barnes & Turner, 2016; Taberlet et al., 2012; Thomsen & Willerslev, 2015). More recently, eDNA approaches have been used for individual identification and population genetics applications (Monge et al., 2020; Wheat et al., 2016). A key benefit of gNIS and eDNA approaches are that they reduce or remove the stress and harm that can come from invasive sampling. Individuals and populations that are monitored with non‐invasive approaches remain relatively undisturbed (Zemanova, 2020). This is particularly valuable for rare or vulnerable species (Storer et al., 2019). Successful gNIS or eDNA sampling has been described across a wide range of species, including mammals (De Barba et al., 2010; Padgett‐Stewart et al., 2016), birds (Miño & Del Lama, 2009; Neice & McRae, 2021), reptiles (Hu & Wu, 2008), amphibians (Eiler et al., 2018; Olson et al., 2012), fish (Jerde et al., 2019; Lieber et al., 2013), and insects (Storer et al., 2019; Uchida et al., 2020).

There are also documented limitations to using non‐invasive samples for genetic analysis (Taberlet et al., 1999). DNA from non‐invasive samples is often of poor quality due to environmental degradation (e.g., ultra‐violet radiation, moisture, and heat), leading to reduced genotyping accuracy, lower loci call‐rates, increased null alleles (allelic dropout), and fewer informative markers (Valière et al., 2007). Whilst the downstream effects of using DNA extracted from non‐invasive samples on population genetic analyses are well understood for mitochondrial and microsatellite markers (McKelvey & Schwartz, 2004; Taberlet et al., 1999), much less is known about the limitations of high resolution next‐generation‐sequencing techniques (e.g., single nucleotide polymorphism [SNP] genotyping) (but see Giangregorio et al., 2019; Schultz et al., 2018). eDNA also experience similar limitations regarding DNA quality (Furlan & Gleeson, 2017; Goldberg et al., 2016; Klymus et al., 2020).

Of the possible factors associated with gNIS which can impact downstream population and individual genetic measures, here we test the impact of three of the most common, namely, DNA degradation, incomplete population sampling, and a reduced SNP panel. We use high‐quality genetic data derived from tissue or blood samples from 430 koalas (>85% of known free‐ranging population) to simulate expected genotypes found in degraded samples from experimental and field studies of non‐invasive koala scat sampling.

The koala (*Phascolarctos cinereus*) is a species for which genetic non‐invasive monitoring of populations could provide a powerful tool. Koalas are listed as “vulnerable” by the IUCN and by national law in the northern parts of their range (Commonwealth of Australia, 2013; Woinarski & Burbidge, 2016) where populations have undergone substantial declines in the past few decades (Rhodes et al., 2015). Koalas face a number of threats, including habitat destruction and fragmentation (Beyer et al., 2018) resulting in small populations with decreased connectivity (Lee et al., 2010). Furthermore, koalas appear unable to avoid mating with closely related conspecifics (Schultz et al., 2020), and island koala populations show evidence of inbreeding depression (Cristescu et al., 2009; Seymour et al., 2001), suggesting that small and isolated populations may be vulnerable to increased risk of inbreeding. Koalas are cryptic in nature and generally occur at low densities, with an estimated average of <1 koala per hectare in southeast Queensland (Rhodes et al., 2015) and densities as low as 0.01 koala per hectare in southwest Queensland (Sullivan et al., 2002). Koala presence‐absence surveys therefore often rely on non‐invasive sampling of scats (Cristescu, Scales, et al., 2018; Jiang et al., 2020), while developments in detection dog use are increasing koala scat survey accuracy, speed, and accessibility (Cristescu et al., 2015). Aside from presence–absence surveys, koala scat can be used for genetic sampling (Schultz et al., 2018; Wedrowicz et al., 2013), making genetic non‐invasive monitoring a feasible tool.

We predict that DNA degradation and reduced SNP panel will result in reduced accuracy and precision of genetic measures, and will require higher population sampling intensity to achieve results comparable to high‐quality data from blood or tissue sampling. Furthermore, we predict that individual‐level genetic measures will be more affected by degraded DNA than population‐level measures.

## 2 MATERIALS AND METHODS

### 2.1 Data collection

This study used koala genetic samples from the Moreton Bay Rail Koala Tagging and Monitoring Program, a long‐term (2013–2017) koala monitoring study that was part of a rail infrastructure development project in southeast Queensland, Australia (−27.234°; 153.036°). During the project, the study area was extensively surveyed, and all identified koalas were captured for veterinary examination and the attachment of tracking devices. Full protocols are available in the project technical report by Hanger et al. (2017). Scientific permits and ethics approvals for catching, handling, veterinary examination and treatment, and monitoring of koalas as follows: Scientific research permits issued by Queensland Department of Environment and Heritage Protection WISP‐11525212, WISP‐16125415, WISP‐13661313, WITK‐14173714, WISP‐17273716; animal ethics approvals from Queensland Department of Agriculture and Fisheries CA‐2012/03/597, CA‐2013/09/719, CA‐2014/06/777, CA‐2015/03/852, and CA‐2016/03/950.

Genetic samples were collected during veterinary examinations and were either blood samples (stored at −20°C) or tissue samples collected during ear tag attachment (stored in 70% ethanol). DNA was extracted using the DNeasy Blood and Tissue Kit (QIAGEN), following the manufacturer's protocol, and DNA extracts were stored at −80°C.

### 2.2 Genotyping and quality control

The SNP dataset used in this study is the same dataset of 8649 SNPs for 430 individuals used in Schultz et al. (2020). SNP genotyping was conducted as per Schultz et al. (2018) and Kjeldsen et al. (2018) by Diversity Arrays Technology, Canberra, using their proprietary DArTseq™ technology. DArTseq™ uses a combination of next‐generation sequencing platforms and DArT complexity‐reduction methods (Courtois et al., 2013; Cruz et al., 2013; Kilian et al., 2012). This process has been well documented in Melville et al. (2017), Lal et al. (2017), and Kjeldsen et al. (2018). Read depth filtering averages in DArTseq pipeline were set at three reads for reference allele, two reads for alternate.

All genetic data sets used in this study were filtered using the dartR package (Gruber et al., 2018), based on the filtering parameters for koala genomics from Kjeldsen et al. (2018) as follows. Loci were included if call rate was greater than 70%, minor allele frequency was greater than 1%, and loci reproducibility (technical replicates) was greater than or equal to 95%. In addition, secondary SNPs (subsequent SNPs on same contig) were removed. This filtered dataset contained 6615 SNP loci and is termed our complete blood/tissue SNP dataset hereafter.

### 2.3 Testing the effects of DNA degradation from non‐invasive sampling on genetic estimates

Genetic non‐invasive sampling typically has two main consequences: (1) DNA quality is lower than when sampling tissue or blood and (2) an unknown proportion of the population is sampled. To test the accuracy of using scat sampling for genetic monitoring of koala populations, we first subsampled our complete blood/tissue SNP dataset and simulated lower call‐rates to replicate genotyping results that one might expect from degraded DNA found in scats. Second, we investigated how the proportion of the population that is sampled influences the accuracy of genetic measures when using non‐invasive sampling.

#### 2.3.1 DNA degradation and sampling intensity

We simulated datasets to reflect SNP data that may be obtained using non‐invasive sampling. To do this, we first randomly subsampled 1300 SNPs from our complete blood/tissue dataset. We selected this number firstly because 1300 loci have been successfully sequenced from experimentally aged koala scats (Schultz et al., 2018) and secondly, we have successfully sequenced similar numbers of SNPs from a field study (see below, Cristescu, Hohwieler, et al., 2018). After subsampling the SNP panel once, we maintained this panel for all subsequent simulations, as randomly subsampling a different panel of 1300 SNPs for each simulation would have introduced variation in genetic measures from randomly including more‐ or less‐informative loci in each simulated SNP panel. This would have introduced variation in genetic estimates due to simulation design, and potentially confounded the effects which we aimed to test here.

**FIGURE 1:**
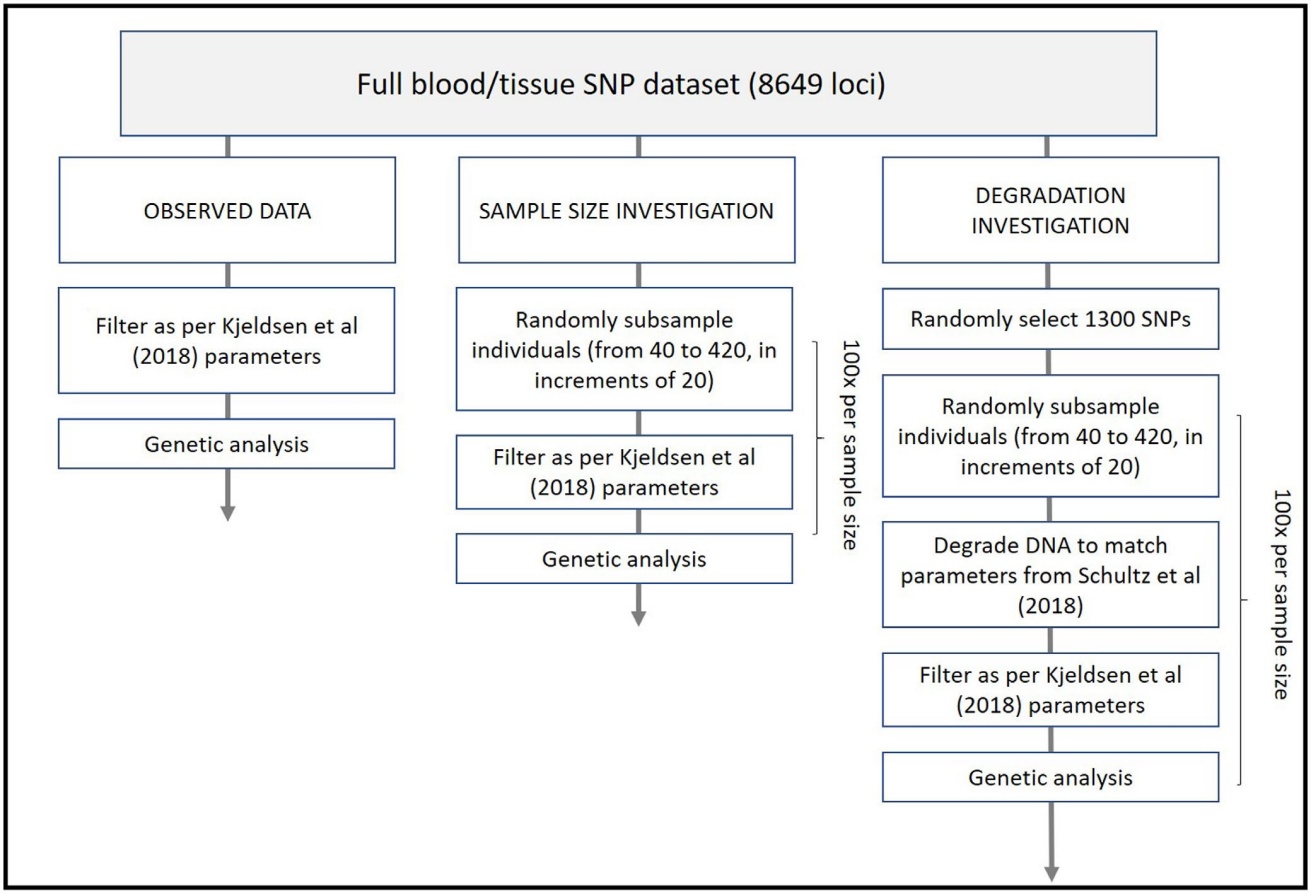
Flowchart of data processing, subsampling, degradation, and analysis

We then subsampled individuals from the full population (*n* = 430) to investigate the effects of population sampling intensity. Specifically, we randomly subsampled between 40 and 420 individuals, in intervals of 20. To mirror spatially explicit sampling approaches used in field projects (Cristescu, Scales, et al., 2018), we applied a spatially explicit thinning protocol using the “spsample” function in the *sp* R package (Bivand et al., 2013; Pebesma & Bivand, 2005). This retains the overall spatial distribution of locations while subsampling a user‐specified number of location points. These location points were the coordinates of first capture of each koala (see Appendix S1). Each population subsample size was replicated 100 times (e.g., 40 koalas × 100 replicates, 60 koalas × 100 replicates, etc.). We then randomly degraded each SNP dataset (i.e., each dataset produced by subsampling 1300 SNPs followed by koala population subsampling). To do this, we used call rate parameters (i.e., proportion of individuals genotyped at a locus) derived from 2‐week old experimentally aged scats (minimum call rate = 0.43, maximum call rate = 1, and mean call rate = 0.62 ± 0.13) (Schultz et al., 2018). Practically, we generated an expected call‐rate for each SNP in the simulated dataset such that the global call‐rate parameters for the dataset matched the maximum, minimum, mean, and standard deviation values described above. However, within each simulated dataset, SNPs were randomly selected for degradation such that an individual locus was not degraded in the same way, or to the same extent, across all simulated datasets. These parameters were similar to those derived from a field study using a DArTcap approach for gNIS from koala scats (see Appendix S1) (Cristescu, Hohwieler, et al., 2018). See Figure 1 for a flowchart of this process.

True non‐invasive sampling from free‐ranging populations would likely include the genotyping of multiple replicates from the same sample or individual to assess genotype accuracy. We do not include such a step here, although the DArTseq™ method does include a “technical replicate” value per locus—a measure of locus reproducibility. We acknowledge that genotyping multiple replicate samples may improve genotype accuracy or reduce missing information.

#### 2.3.2 Individual and population genetic measures

For each simulated dataset, we filtered the data as described above and measured the following population and individual genetic measures: expected heterozygosity (*H*_e_), Shannon's information index (*I*), inbreeding coefficient (*F*_IS_) (Kjeldsen et al., 2015; Sherwin et al., 2006), spatial autocorrelation, and internal relatedness (IR). We compared estimates derived from simulated datasets to those from the complete blood/tissue dataset to identify the accuracy and precision of simulation‐derived measures. Here accuracy refers to how close estimated values are to the observed values. We refer to precision as the degree of variability between estimates from replicate simulated datasets. We measured large‐scale genetic structure (population structure) using the package TESS v2.1.0 (Chen et al., 2007) on our complete blood/tissue SNP dataset. However, as we found evidence of only one population, we did not run large‐scale population structure measures again.

Inbreeding coefficient (*F*_IS_) is calculated as:

$$F_{\text{IS}}=\frac{H_{e}‐H_{o}}{H_{e}}$$

where *H*_e_ is expected heterozygosity and *H*_o_ is observed heterozygosity. All genetic measures were analyzed in the R Statistical Environment v3.4.3 (R Core Team, 2018).

We measured fine‐scale genetic structure using spatial autocorrelation analyses as this can be an indicator of inbreeding risk (Banks & Peakall, 2012). This correlates pairwise genetic distance with geographic distance, for different distance classes, and uses a bootstrapping approach to assign significance at different distance classes. This was measured in the R package *PopGenReport* (Adamack & Gruber, 2014). See Appendix S1 for distance class calculations. To augment our spatial autocorrelation analyses, we determined a biologically meaningful maximum pairwise distance at which koalas in the study population may breed. This allowed us to test for fine‐scale genetic structure within this distance to determine whether individuals within the population were at risk of breeding with related conspecifics. The maximum likely breeding distance identified here (see Appendix S1) informed spatial autocorrelation analyses to assess inbreeding risk and accuracy of spatial autocorrelations at different population sizes. *H*_e_ and *F*_IS_ were calculated using the *adegenet* package (Jombart, 2008) and *I* was calculated using the *Poppr* package (Kamvar et al., 2014).

Internal relatedness was calculated as:

$$\text{IR}=\frac{2H‐\sum f_{i}}{2N‐\sum f_{i}}$$

where *H* is the number of loci that are homozygous, *N* is the number of loci, and *f_i_* is the frequency of the *i*th allele contained in the genotype (Amos et al., 2001). IR was measured using the GENHET v3.1 function in the R Statistical Environment (Coulon, 2010). As IR is an individual measure relative to the genotypes of the other individuals sampled in the population, we investigated the correlation between the IR values measured for each individual in each simulated dataset and the actual individual IR calculated from our complete blood/tissue SNP dataset. In this way we assessed whether the patterns of inbreeding found in the observed dataset were accurately identified in the simulated datasets. Here we selected the Pearson correlation coefficient (*r*) due to normally distributed data.

## 3 RESULTS

### 3.1 Genetic analysis of observed population

Our complete blood/tissue SNP dataset consisted of 430 individual koalas from the same population, with 8649 SNP loci prior to filtering, and 6615 loci post‐filtering. From this dataset we estimated population *H*_e_ as 0.284 ± 0.002, *I* as 0.441 ± 0.002, and *F*_IS_ as 0.125. Average internal relatedness (IR) was estimated as 0.12 ± 0.004 (range −0.33–0.43).

**FIGURE 2:**
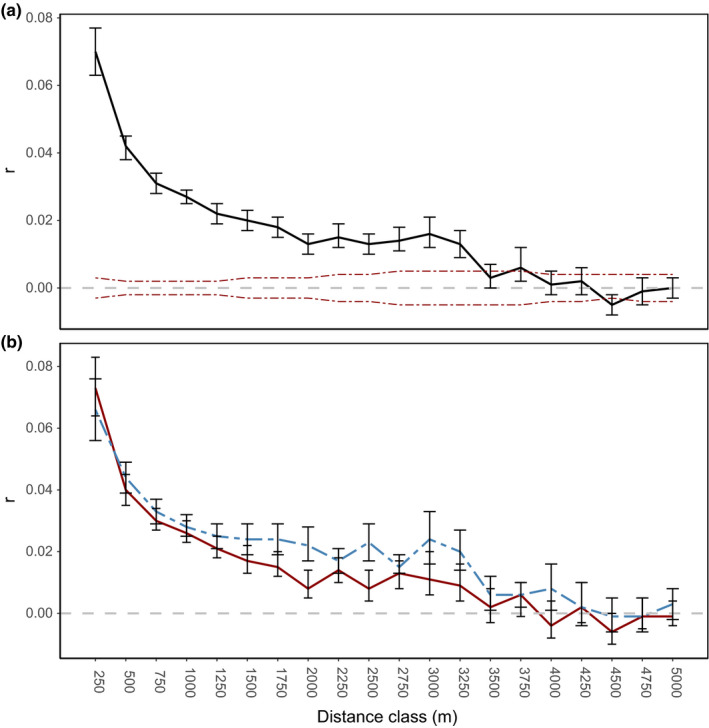
Spatial autocorrelation correlograms of genetic and geographic distance for male, female, and combined koalas in a wild population. Genetic data were generated using 6615 filtered single nucleotide polymorphism loci from blood or tissue samples. Error bars (95% confidence) around the autocorrelation *r* values were generated from 999 bootstrap iterations. (a) Spatial autocorrelation for entire population (*n* = 430), red dashed lines indicate upper and lower bounds of a 95% confidence interval for *r*, generated under null hypothesis of random geographic distribution of koalas. (b) Spatial autocorrelation correlograms for male and female koalas. Dashed line (blue) is male koalas, solid line (red) is female koalas

Using parentage assignment data from Schultz et al. (2020), we estimated that a maximum likely breeding distance between pairs of koalas was 500 m (see Appendix S1 for details). From our complete blood/tissue SNP dataset, we found evidence of fine‐scale genetic structure in the study population (Figure 2a). That is, within each 250‐m distance class until 3500 m, there were more closely related koalas to the focal individual than expected by chance (*p* < .05 derived from bootstrapping). We did not find evidence of sex‐biased dispersal in this population (i.e., male and female koalas within the population showed closely comparable genetic structure) (Figure 2b).

### 3.2 DNA degradation and sampling intensity

**FIGURE 3:**
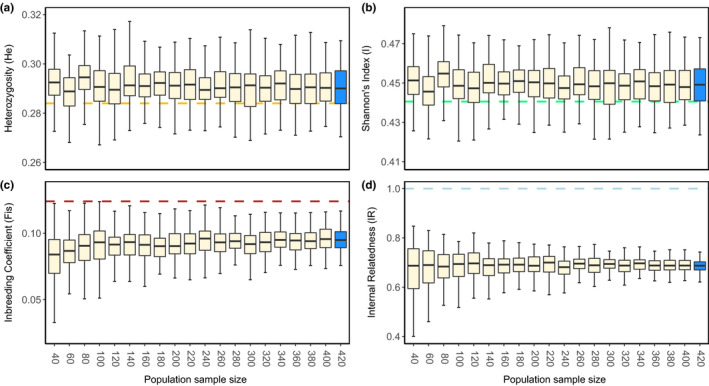
Genetic measures at different sample sizes from simulations degraded to match call rate parameters and single nucleotide polymorphism (SNP) panel from 2‐week old experimentally aged koala scat. (a–c) Population genetic measures (expected heterozygosity, Shannon's information index, inbreeding coefficient) estimates from five replicates at each samples size (40–420 koalas). Dashed line represents actual metric value for total population of 430 koalas, calculated using high quality tissue/blood DNA extracts. (d) Pearson correlation (*r*) between observed internal relatedness, and internal relatedness measures for population subsamples from datasets simulated to match experimentally aged scat call rates. Dotted line represents an exact correlation (*r* = 1). Shaded boxplots represent 420 individuals (98% of population), and so provides information on the variance in analysis outcome due only to DNA degradation and reduced SNP panel

Using simulated datasets, we found that DNA quality affected the accuracy of diversity estimates (Figure 3a,b). Heterozygosity estimates were, on average, overestimated by 0.007, whereas Shannon's information index was overestimated by, on average, 0.01. For both diversity measures, precision was lower (i.e., replicates were more variable) than diversity estimates at corresponding population sample sizes from the complete blood/tissue SNP dataset. We found that population inbreeding coefficient measures were precise, but consistently underestimated. (Figure 3c). For *H*_e_, *I*, and *F*_IS_, we found that precision was not strongly affected by the number of individuals sampled, with large sample sizes having very similar distributions to small sample sizes. At the individual level, we found that simulated and observed IR values still displayed a positive correlation, with an average correlation of *r* = .69 (Figure 3d).

**FIGURE 4:**
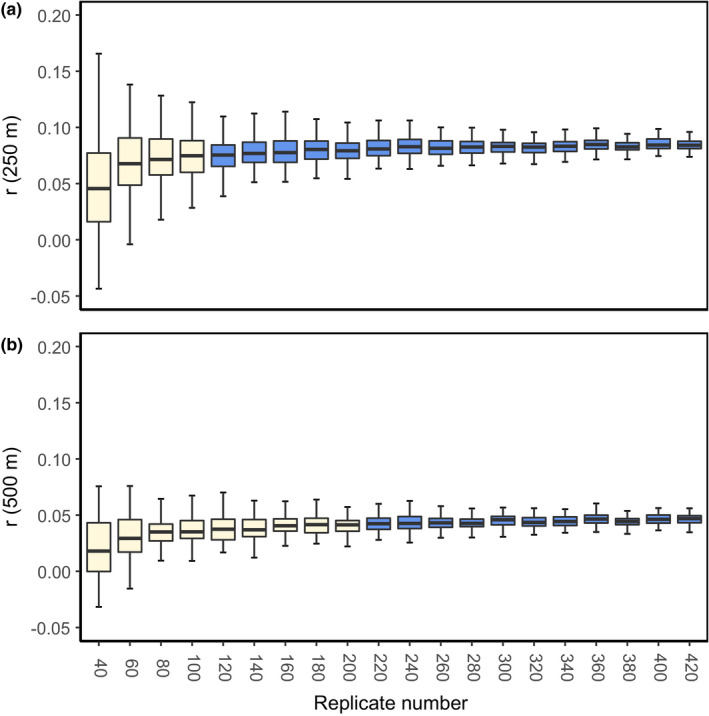
Accuracy of genetic and geographic spatial autocorrelation analyses for degraded DNA at different population sample sizes. Genetic data were generated using from a subset of 1300 single nucleotide polymorphism loci, which were then degraded to match call rate parameters from experimentally aged scat DNA samples. Sample sizes highlighted in blue indicate that >95% of replicates at that sample size displayed positive genetic structure, determined from 999 bootstrap iterations per replicate. Each sample size had 100 simulated replicates. (a) Variance in spatial autocorrelation *r* values at 250‐m distance class. (b) Variance in spatial autocorrelation *r* values at 500‐m distance class

When we assessed the sample sizes required for accurate genetic measures using the simulated datasets, we found that increasing sample size had little effect on improving accuracy or precision for both diversity indices. Similarly, population inbreeding coefficients were underestimated consistently (Figure 3c) at all sample sizes, with a small increase in precision with increasing sample size. We found that accuracy of IR correlation was not affected by sample size, although the precision of IR correlation increased until 140 koalas were sampled (33% of population). Finally, we found significant positive genetic structure at 120 koalas sampled (28% of population) and higher for the 250‐m distance class (Figure 4a), and at 220 koalas (51% of the population) and higher for the 500‐m distance class (Figure 4b). Errors in *r* value reduced as population sample size increased, with a maximum error of 0.14 (40 koalas; 9% of population) for the 250‐m distance class, and 0.04 (40 koalas) for the 500‐m distance class. Errors for the 250‐m distance class fell below 0.04 from 180 koalas (42% of population) onwards, and for the 500‐m class below 0.02 from 220 koalas (51% of population) onwards.

We also found variation in population genetic measures due only to degradation of differentially informative SNPs across replicates (see highlighted 420 individuals sample sizes, Figure 3). This highlights how degrading the same SNP set in different ways can impact the precision of analyses, even at extremely high sample sizes (98% of population).

We also conducted simulations investigating the impact of sampling intensity without genetic degradation (see Appendix S1 for methods and results). We found that the proportion of population sampled had negligible impact on most genetic measures when high‐quality DNA was used.

## 4 DISCUSSION

For wildlife managers hoping to conduct genetic monitoring of at‐risk populations, the best available genetic data may often be from genetic non‐invasive samples. Such data typically display reduced call rates and SNP panel, and likely also come from incomplete population sampling (Russello et al., 2015). Basing management decisions on incomplete genetic datasets can have negative implications for conservation outcomes, particularly given the limited funding available for conservation management (Waldron et al., 2013). When incomplete or inaccurate genetic datasets are used in conservation decision‐making, much needed interventions may be overlooked, resulting in population genetic impacts such as increased inbreeding, reduced geneflow, or both (Kenney et al., 2014), and the potential‐associated fitness reductions (Edmands, 2007) may follow.

We found that simulated genetic samples, even with the reduced call rates and SNP panel typical of non‐invasive sampling, can provide useful and informative genetic estimates for free‐ranging populations. By sampling more than 60 koalas (14% of population), we achieved consistent but slightly overestimated diversity measures (*I* and *H*_e_), and precise but underestimated inbreeding coefficients (*F*_IS_). For internal relatedness, precision was achieved when 140 individuals were sampled (33% of population), while accuracy was not strongly influenced by sample size and showed moderate to strong correlations with true IR values (average = 0.69). For accurate spatial autocorrelation estimates using non‐invasive data, which can be used to assess inbreeding risk in free‐ranging populations, 28% of the population required sampling to find positive fine‐scale genetic structure (matching that found in the complete blood/tissue dataset) at 250 m, and 51% of the population at 500 m.

That low samples sizes (e.g., 14% of the population) can provide reasonably accurate diversity and inbreeding coefficients suggests that practitioners may be able to design studies with less sampling intensity than perhaps anticipated. While this may increase the cost‐effectiveness of gNIS of populations, it is important to acknowledge that these sample sizes are based on a single population of koalas, and may not translate across other species and study systems. It is therefore important for practitioners to engage with their own study systems to determine sufficient sampling intensities.

Although we found that population *F*_IS_ was consistently underestimated, we found that inbreeding coefficients were very precise in their underestimation. Inbreeding estimates in conservation are often a baseline measure used to inform management decisions, particularly interventions such as genetic rescue or facilitating increased connectivity (Benson et al., 2016; Hedrick & Fredrickson, 2009). In koala populations, *F*_IS_ values have been documented to vary greatly between populations and regions (e.g., 0.09 [Cristescu et al., 2011] to 0.32 [Kjeldsen et al., 2015]). Despite these underestimations, inbreeding coefficient values from non‐invasively collected samples can still provide valuable information for conservation planning.

While no clear‐cut constant inbreeding thresholds exist (below which conservation interventions are required), genetic rescue decisions are made using a wide range of data, not only inbreeding coefficients (Hedrick & Fredrickson, 2009). Furthermore, at the landscape scale where multiple populations are assessed using standardized SNP panels and techniques, consistent underestimations of inbreeding across populations may still provide useful information on relative population inbreeding levels and identify areas of higher or lower inbreeding. Populations with relatively higher inbreeding levels could then be reassessed with higher‐density markers to identify which regions of the genome are being depleted by inbreeding.

In this study, the consistent underestimation of inbreeding coefficient values and overestimation of diversity indices is likely due to insufficient markers, as around 5000 SNPs are generally required for accurate genome‐wide diversity estimates (Benjelloun et al., 2019). The utility of non‐invasive sampling for population inbreeding assessment is therefore dependent on the specific questions being asked, and caution should be taken when interpreting or using inbreeding coefficients calculated from gNIS to determine population genetic health.

Similarly, although raw IR values from the simulated datasets differed from observed IR values, the moderate to high correlations between observed and simulated values implies that relative differences between individuals’ IR values are maintained when using non‐invasively collected samples. This is encouraging for researchers interested in patterns of individual‐level genetic measures, such as those linking individual‐level inbreeding to fitness measures (Acevedo‐Whitehouse et al., 2003) or investigating relatedness and estimating pedigrees (Hedmark & Ellegren, 2007).

We also found that positive fine‐scale genetic structure is correctly identified by non‐invasive sampling simulations when 120 koalas (28% of population; for 250‐m distance class) or 220 koalas (51% of population; for 500‐m distance class) are sampled. Accurate spatial autocorrelation measures therefore require higher sampling effort than genetic diversity measures but may help to inform inbreeding risk where population inbreeding coefficient is not sufficient to identify whether intervention is required.

It is important to note some of the limitations to our study. Here we have tested some of the more common factors associated with gNIS—namely reduced SNP panel, reduced call rates, and variable population sampling size. However, there may be other impacts on genotype accuracy caused by degraded DNA from non‐invasive samples. Null alleles, where heterozygous loci are incorrectly read as homozygous due to allelic dropout, is one example of this, and the degree of allelic dropout can vary between samples (Schultz et al., 2018). Allelic dropout seems associated with scat sampling for DNA in particular (Stenglein et al., 2010), although certain DNA extraction methods may reduce this prevalence (Vynne et al., 2012). We have not included simulations of allelic dropout in this study, but it is a known drawback of using non‐invasive samples, and warrants further investigation. Depending on the method of non‐invasive sampling used (e.g., scat, hair, and eDNA), the DNA of multiple individuals may be collected as a single sample, and care is needed in survey design and DNA processing to account for this (Roon et al., 2005). Similarly, miscalling of SNPs due to low DNA quality can result in “ghost” individuals, where inaccurate repeated genotyping of the same individual results in the genetic identification of non‐existent “individuals” in the population (Lampa et al., 2015). Guidelines exist for dealing with such issues in genetic capture‐mark‐recapture studies, and gNIS has already shown promise in this area (Sabino‐Marques et al., 2018), but further investigation is required for other applications (Lampa et al., 2013). Nevertheless, the results we present contain important information about the impacts of SNP panel size, call‐rates, and sampling intensity on downstream genetic metrics.

As per our predictions, we found that the DNA degradation of non‐invasive samples reduced accuracy and precision of genetic measures, and some measures required higher sampling intensity to achieve useable results. However, the overestimation of diversity measures and underestimation of inbreeding coefficients was not anticipated. Our results provide strong evidence that next‐generation sequencing data from non‐invasively sampled DNA can be an effective tool for genetic monitoring, provided adequate attention is given to the limitations identified. By acknowledging such limitations where necessary, the degree of accuracy and precision attainable through gNIS implies that wildlife managers can use such data to guide both non‐genetic (e.g., rehabilitation of movement corridors) and genetic (e.g., genetic rescue or translocations) conservation interventions (Schwartz et al., 2007). Furthermore, the results of this study, particularly the consistent under‐ and over‐estimation of diversity measures and inbreeding coefficients respectively, strongly suggests that SNP panel size, and the degree of variation contained in those SNPs, will interact with DNA degradation and population sample size to impact downstream genetic analyses. Future research investigating these interactions, particularly across a variety of species, may provide generalizable rules for planning gNIS.

The accuracy and utility of non‐invasive sampling can be further maximized by developing a targeted SNP panel for the focal species, allowing for repeatable genotyping of the same loci across populations/regions, as required for population comparisons and landscape‐level investigations. Here 1300 SNPs was sufficient for the chosen analyses, but requirements for other non‐invasive approaches and species may differ, particularly as assessing the required number of SNPs will depend on the amount of variation found in those SNPs (see Morin et al., 2004; Smouse, 2010; Strucken et al., 2016). Although some application of gNIS will necessarily rely on very low‐quality samples yielding only a few hundred SNPs (Natesh et al., 2019; Schmidt et al., 2020), other published studies use >500 SNPs (Janjua et al., 2020) while others use whole genome sequencing approaches from non‐invasive samples (Khan et al., 2020). Recent developments in microfluidic genotyping (von Thaden et al., 2017) which allow for rapid genotyping of multiple samples using reduced SNP panels (hundreds of SNPs) will also influence the accessibility of SNP genotyping for non‐invasive samples. Guidelines now exist for practitioners to develop reduced SNP panels for use in non‐invasive genotyping using microfluidic approaches, suggesting an imminent increase in the use of such technologies for ongoing monitoring of vulnerable species (von Thaden et al., 2020).

Finally, the results of this study suggest that low sample numbers and smaller SNP panels can provide accurate downstream genetic information. Coupled with ongoing reductions in costs of next‐generation sequencing approaches (Ferreira et al., 2018; Monterroso et al., 2019), gNIS will increasingly provide cost‐effective methods for genetic monitoring. We anticipate that such sampling will become more widespread and accessible, and so studies such as this which investigate the downstream impacts of such sampling on genetic analyses will therefore become increasingly helpful in guiding future monitoring.

## CONFLICT OF INTEREST

The authors declare no conflict of interest.

## AUTHOR CONTRIBUTIONS

**Anthony James Schultz:** Conceptualization (equal); Formal analysis (equal); Investigation (lead); Methodology (supporting); Project administration (supporting); Writing – original draft (lead); Writing – review & editing (lead). **Kasha Strickland:** Formal analysis (lead); Investigation (equal); Methodology (lead); Software (lead); Validation (lead); Writing – original draft (supporting); Writing – review & editing (equal). **Romane H. Cristescu:** Investigation (equal); Methodology (equal); Resources (supporting); Supervision (supporting); Writing – review & editing (equal). **Jonathan Hanger:** Data curation (lead); Project administration (equal); Resources (equal); Writing – review & editing (equal). **Deidre de Villiers:** Data curation (lead); Project administration (equal); Resources (equal); Writing – review & editing (equal). **Céline H. Frère:** Conceptualization (lead); Funding acquisition (lead); Investigation (equal); Project administration (supporting); Resources (equal); Supervision (lead); Validation (equal); Writing – original draft (equal); Writing – review & editing (equal).

## Supporting information

Appendix S1

## Data Availability

The de‐identified data and R programming code used in this study are available on Dryad (https://doi.org/10.5061/dryad.1ns1rn8vq) and GitHub (https://github.com/Anthony‐Schultz/Non‐Invasive‐DNA‐Testing.git).
