# Supplementary material for: Testing the effectiveness of genetic monitoring using genetic non‐invasive sampling
Source: Ecol Evol. 2021 Dec 27;12(1):e8459. doi: 10.1002/ece3.8459 (PMC8794716; doi:10.1002/ece3.8459)
Supplement: Supplementary file 1 — Appendix S1 [file ECE3-12-e8459-s001.docx]

SUPPLEMENTARY INFORMATION

Non-invasive genetic sampling of koala scat in field study, and DArTcap method

The call rate metrics and SNP panel size used in this project were derived from experimental scat ageing in the laboratory (Schultz et al., 2018), and were similar to those generated by a field study conducted by this research group (Cristescu, Hohwieler, et al., 2018). In this field study, 431 koala scats from a free-ranging population were genotyped using a DArTcap approach conducted by Diversity Arrays, Canberra. DArTcap technology is similar to the DArTseq^TM^ approach used in this study, but applies a selective step after complexity reduction, whereby specific markers are genotyped. This approach uses nucleic acid probes which bind to the restriction fragments which carry the target markers. The restriction fragments are enriched during this capture step, resulting in higher sequencing (and so genotyping) success. This DArTcap dataset had 1088 SNPs with a median call rate = 0.79 (minimum = 0.49, maximum = 1). This similarity in number of loci and call-rate parameters suggest that those used here are comparable to field studies using non-invasive genetic approaches.

Geographic locations of koalas

All koalas in the study were fitted with VHF and/or GPS tracking attachments. Koala tracking schedules varied across the study, but typically koalas with VHF attachments were tracked twice per week, whereas koalas with GPS attachments had locations recorded twice per day. For population genetic analyses which required spatial coordinates, we used the first tracking point recorded for each koala, typically the point where each individual was captured for its initial veterinary examination and tracking device attachment. Selecting the first recorded tracking point allowed us to include individuals which were euthanised post-capture due to pre-existing illness or injury, provided a genetic sample was collected from them. Furthermore, using the first tracking point over a calculated centre-of-activity meant that translocated koalas were geographically positioned in the area of their original capture, rather than a confounded centre-of-activity averaged across pre- and post-translocation tracking points. We also removed koalas from this study who were still dependent joeys when their mothers were translocated during development of the infrastructure project. Joeys were typically fitted with tracking devices at independence (9 – 11 months old), thus we risked recording a joey’s first tracking point in the region its mother had been translocated to, not the region it was conceived and born in. This geographic / genetic mismatch may have confounded our analyses, and so these joeys were removed.

Spatial autocorrelation distance classes

We selected a distance class size of 250 m, with 20 classes from zero to 5000 m. We trialled distance classes from 100 m upwards (in increments of 50 m) and this was the smallest distance for which each size class contained sufficient individuals (> 5) to estimate genetic structure, even at very low population sampling sizes. We did not investigate more complex applications of spatial autocorrelation analyses (i.e. selecting only male-female pairs) which may provide additional information on inbreeding risk (Blyton et al., 2015), as the aim of this paper is to test the impact of using non-invasively sampled DNA on genetic measures, not exhaustively investigate the population genetics of the study population.

Maximum likely koala breeding distance calculation

We used parentage assignment data and VHF tracking locations from a previous study (Schultz et al., 2020) to determine pairwise geographic proximities at which successful koala mating events occurred. These distances were calculated by measuring the pairwise distances between the centres of activity of fourteen known breeding koala pairs. Centres of activity were calculated from VHF tracking points over a period of two months straddling (28 days before, 28 days after) each pair’s estimated successful copulation date leading to conception.

We found that the distances between centres of activity for fourteen known breeding pairs ranged from 73 m to 410 m, with a mean of 159 m ± 99 m. Given that twice weekly VHF tracking is unlikely to capture the exact moment of copulation between pairs, particularly if copulation occurs at night, these distances provide an estimation of the average distance between individuals around the time of successful copulation. That is, successful mating between males and females may occur when individuals are, on average, less than 500m from each other

Investigating impact of sampling intensity with high quality DNA samples on genetic measures

Methods

To complement our simulations on the impact of DNA degradation and sampling intensity on genetic measures, we also conducted similar simulations where we maintained the complete tissue/blood dataset for our full population (i.e. we did not subsampled SNP panel or alter call-rate parameters) and only subsampled proportions of the population. This was to investigate how sampling intensity alone can affect genetic measures. To do this we randomly subsampled individuals from the complete tissue/blood SNP dataset at increasing increments. Specifically, using the complete tissue/blood SNP dataset of our observed population (n = 430), we randomly subsampled between 40 and 420 individuals, in intervals of 20. In order to mirror sampling approaches used in field projects, which often focus on obtaining an even geographic spread of samples across the study site (Cristescu, Scales, et al., 2018), we subsampled individuals using a spatially-explicit thinning protocol using the “spsample” function in the *sp* R package (Bivand et al., 2013; Pebesma & Bivand, 2005). This function retains the overall spatial distribution of points while subsampling a user-specified number of location points. These location points were the coordinates of first capture of each koala. Each subsample size was replicated 100 times.

Results

In comparison to our complete blood/tissue SNP dataset, we found that for both diversity indices (*I* and *H_e_*), accuracy was variable when sampling below 23% of the population (i.e. 100 individuals) but highly accurate above this. However, at these low population sizes (< 23%) the deviation from the observed value was small (Figure Supp. 1a, 1b), resulting in functionally accurate estimates at all population sizes. For both measures, increasing the sampling size generally improved precision of estimates, as differences between observed and simulated diversity measures remained small. H*_e_* measures had a maximum error of 0.007 across sample sizes, whereas *I* had a maximum error of 0.009. The precision of population F*_is_* measures increased with sample size, although the maximum error was still low (0.04) and high accuracy (maximum error of 0.02 or less) was first achieved at 23% of population sampled / 100 koalas, with a maximum error 0.016. F*_is_* errors then remain below 0.02 for all sample sizes (Figure Supp. 1c). We also found that *IR* estimates correlated almost exactly (*r* > 0.99) for all subsampling sizes (Figure Supp. 1d). It is important to note that the y-axis scales in the Supplementary Figures do not match the scales used in the Main Text figures, and this should be taken into account when comparing results. We found significant positive genetic structure (matching the patterns seen in the complete blood/tissue dataset) at 80 koalas (19% of population) and higher for the 250 m distance class (Figure Supp. 2a) and 120 koalas (28% of population) and higher for the 500 m distance class (Figure Supp. 2b). Errors in *r* value reduced as population sample size increased, with a maximum error of 0.1 (60 koalas; 14% of population) for the 250 m distance class, and 0.04 (40 koalas; 9% of population) for the 500 m distance class. These errors quickly fell, with errors for the 250 m distance class falling below 0.04 from 200 (47% of population) koalas onwards, and for the 500 m class below 0.02 from 140 (33% of population) koalas onwards.


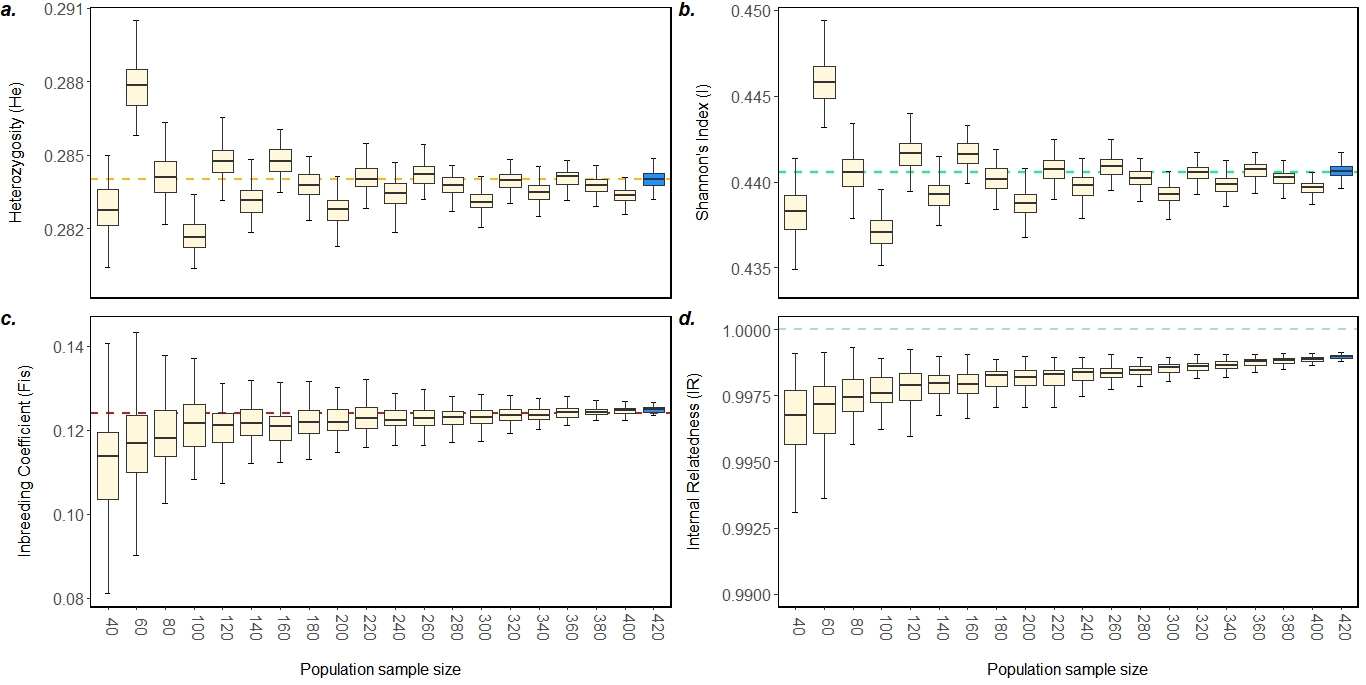


**Figure** **Supplementary** **1**. Genetic measures at different population subsampling sizes. Genotyping data from blood or tissue DNA extraction from wild koala population. **(A)** to **(C):** Population genetic measures (Expected heterozygosity, Shannon’s information index, inbreeding coefficient) estimates from 100 replicates at each samples size (40 to 420 koalas). Dashed line represents actual metric value for total population of 430 koalas, calculated using high quality tissue/blood DNA extracts. **(D)**: Pearson correlation (*r*) between full observed internal relatedness (n=430) and internal relatedness measures for population subsamples. Dotted line represents an exact correlation (*r* = 1). Shaded boxplots represent 420 individuals (98% of population), and so provides information on the variance in analysis outcome due only to variance in the population.


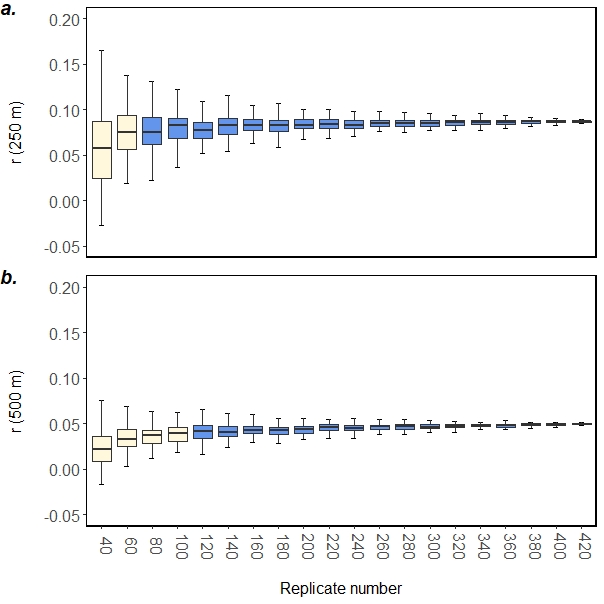


**Figure Supplementary 2.** Accuracy of genetic and geographic spatial autocorrelation analyses for different population sample sizes. Genetic data were generated using 8649 SNP loci from blood or tissue samples. Sample sizes highlighted in blue indicate that > 95% of replicates at that sample size displayed positive genetic structure, determined from 999 bootstrap iterations per replicate. Each sample size had 100 simulated replicates. **(A)** Variance in spatial autocorrelation *r* values at 250 m distance class. **(B)** Variance in spatial autocorrelation *r* values at 500 m distance class.

References

Bivand, R. S., Pebesma, E. J., & Gomez-Rubio, V. (2013). *Applied spatial data analysis with R: Vol. http://www.asdar-book.org* (Second Edition). Springer.

Blyton, M. D. J., Banks, S. C., & Peakall, R. (2015). The effect of sex-biased dispersal on opposite-sexed spatial genetic structure and inbreeding risk. *Molecular Ecology*, *24*(8), 1681–1695. https://doi.org/10.1111/mec.13149

Cristescu, R. H., Hohwieler, K., Strickland, K., Littleford-Colquhoun, B. L., & Frere, C. (2018). *Final Report: Redland Coast Koala Population Assessment Project*.

Cristescu, R. H., Scales, K. L., Schultz, A. J., Miller, R. L., Schoeman, D. S., Dique, D., & Frère, C. H. (2018). Environmental impact assessments can misrepresent species distributions: A case study of koalas in Queensland, Australia. *Animal Conservation*. https://doi.org/doi:10.1111/acv.12455

Pebesma, E. J., & Bivand, R. S. (2005). Classes and methods for spatial data in R. *R News*, *5*(2). https://cran.r-project.org/doc/Rnews/

Schultz, A. J., Cristescu, R. H., Hanger, J., Loader, J., de Villiers, D., & Frère, C. H. (2020). Inbreeding and disease avoidance in a free-ranging koala population. *Molecular Ecology*, *29*(13), 2416–2430. https://doi.org/10.1111/mec.15488

Schultz, A. J., Cristescu, R. H., Littleford-Colquhoun, B. L., Jaccoud, D., & Frere, C. H. (2018). Fresh is best: Accurate SNP genotyping from koala scats. *Ecology and Evolution*, *8*(6), 3139–3151. https://doi.org/10.1002/ece3.3765
